# Dapagliflozin’s Role in Heart Failure: An Overview of Clinical Trial Evidence

**DOI:** 10.7759/cureus.70727

**Published:** 2024-10-02

**Authors:** Rizwan H Khan, Hasham Qureshi, Zubair Ahmed, Amir Hassan, Syed Ahsan Ali, Sagar Kumar, Aasia Ismail, Nouman Anthony, Muhammad Murtaza M Shabbir, Umar Azam Ali, Najeeb Ullah, Ibrar Khaliq

**Affiliations:** 1 Internal Medicine, Northern Ireland Medical and Dental Training Agency, Belfast, GBR; 2 Psychiatry, Killester Mental Health Services, Dublin, IRL; 3 Internal Medicine, Mersey and West Lancashire NHS Trust, Walsall, GBR; 4 Internal Medicine, Shaukat Khanum Memorial Cancer Hospital and Research Center, Peshawar, PAK; 5 Cardiology, Nottingham University Hospitals NHS Trust, Nottingham, GBR; 6 Internal Medicine, Health Department Government of Sindh, Mithi, PAK; 7 Internal Medicine, Jinnah Postgraduate Medical Center, Karachi, PAK; 8 Internal Medicine, Rehman Medical Institue, Peshawar, PAK; 9 Emergency Medicine, Hameed Latif Hospital, Lahore, PAK; 10 Internal Medicine, Ayub Medical College, Abbottabad, PAK; 11 Internal Medicine, Rehman Medical Institute, Peshawar, PAK; 12 Internal Medicine, Services Hospital Lahore, Lahore, PAK

**Keywords:** dapagliflozin, efficacy, heart failure, sglt2 inhibitors, systematic review

## Abstract

Heart failure (HF) has become a global health threat, necessitating the development of novel treatment options to address this crisis. Sodium-glucose co-transporter-2 (SGLT2) inhibitors, such as dapagliflozin, may offer significant advantages in the treatment of HF, particularly in patients with reduced ejection fraction (HFrEF). This systematic review combines the findings from clinical studies to evaluate the safety and effectiveness of dapagliflozin in HF patients. The study results demonstrate dapagliflozin's consistent improvements in reducing adverse cardiovascular events, including cardiovascular death rates across different HF phenotypes. Mechanistically, dapagliflozin exerts diuretic and hemodynamic effects, along with its actions on myocardial metabolism, ion transporters, fibrosis, adipokines, and vascular function, which collectively contribute to its cardioprotective effects. This overview emphasizes dapagliflozin’s key role in HF management, positioning it as one of the central pillars of the HF treatment regimen. Further studies are essential to fully understand dapagliflozin’s long-term effectiveness, safety, and integration into routine clinical practice, ultimately improving patient outcomes in HF.

## Introduction and background

Heart failure (HF) is a significant clinical challenge worldwide, affecting over 64 million people and placing a considerable burden on healthcare systems globally [1]. Among the various subtypes of HF, HF with reduced ejection fraction (HFrEF) represents one of the most challenging to manage due to its association with higher rates of morbidity and mortality [2]. Guideline-directed medical therapy (GDMT), historically focused on blocking neurohormonal pathways that lead to adverse cardiac remodeling, has evolved significantly over the past few years [3]. One of the notable advances has been the introduction of sodium-glucose co-transporter-2 (SGLT2) inhibitors, initially developed for type 2 diabetes mellitus (T2DM), but now recognized for their efficacy in managing HF, particularly in patients with HFrEF [4,5].

Recent clinical trials, such as the DAPA-HF and DELIVER studies, have demonstrated strong evidence supporting the effectiveness of SGLT2 inhibitors like dapagliflozin in reducing HF hospitalizations and cardiovascular (CV) events, which are major independent outcomes in HF management [6,7]. These trials highlight dapagliflozin's potential to improve outcomes in both HF with preserved ejection fraction (HFpEF) and HFrEF, irrespective of its glucose-lowering properties [8]. Given the increasing body of evidence supporting dapagliflozin's role in HF treatment, there is a need for a comprehensive systematic review to consolidate recent clinical data into one document [9].

This systematic review evaluates the effectiveness and safety of dapagliflozin in individuals with HF by analyzing its impact on key outcomes such as mortality, HF hospitalizations, CV events, and changes in functional status and quality of life. Additionally, we explore the potential mechanisms underlying dapagliflozin’s beneficial effects and assess its safety profile. Through a rigorous synthesis of the available evidence, this review aims to provide clinicians and researchers with valuable insights into the role of dapagliflozin as a therapeutic agent in managing HF. The review seeks to address gaps in knowledge regarding dapagliflozin's use for HF treatment and to offer a practical guide by systematizing data from multiple clinical trials.

## Review

Materials and methods

Search Strategy

This study aims to review the effectiveness of dapagliflozin in HF patients by systematically analyzing available clinical evidence. Meta-analyses and systematic reviews are crucial in providing reliable and consistent reports on healthcare interventions, as they combine data from multiple studies to generate more robust conclusions and minimize biases [9]. To ensure the accuracy and comprehensiveness of this review, we followed the Preferred Reporting Items for Systematic Reviews and Meta-Analyses (PRISMA) guidelines [10]. PRISMA provides a structured and transparent process for conducting systematic reviews, ensuring that data is collected and analyzed in a scientifically rigorous and consistent manner.

A thorough examination of the literature was conducted using established online databases, including PubMed, Google Scholar, and Scopus. The search focused on studies related to the management of HF and the use of dapagliflozin. Ethics committee approval was deemed unnecessary for this systematic review, as it synthesized previously published studies.

Eligibility Criteria

The search strategy combined terms related to cardiac emergencies, resuscitation techniques, and advancements in CV care with medical topic headings (MeSH) terminology. These keywords indicate the relationship intended to achieve the goals of the research. Boolean operators were added to the carefully chosen and organized keywords and phrases to improve search efficiency. To gather data, these terms had to be searched inside the “articles” of the online dataset. It was necessary to compile a list of papers that had comparable terms in their abstracts, article titles, and keywords - that is, their author keywords and indexed keywords. The publishing range of 10 years was first taken into consideration; however, to include the most recent changes only, subsequently, it was limited to the issues released between 2019 and 2024. These aforementioned studies were published in credible journals, entailing only seven articles for this systematic review. Table 1 shows the inclusion and exclusion criteria for the most relevant articles.

**Table 1:** A summary of the inclusion and the exclusion criteria.

Criteria	Inclusion	Exclusion
Publication Year	Papers published between 2019 and 2023.	Papers published before 2019.
Study Type	Peer-reviewed articles, systematic reviews, meta-analyses, and original research studies.	Editorials, commentaries, opinions, and non-peer-reviewed articles.
Subject	Studies focusing on dapagliflozin's effectiveness and safety in individuals with HF with a focus on the effects on HF hospitalization, cardiovascular events, mortality, and other related endpoints.	Studies not specifically addressing use of dapagliflozin in individuals with HF.
Language	Articles published in English.	Articles published in languages other than English.
Data Availability	Studies with accessible and clear data/results.	Studies with inaccessible data or unclear results.
Methodology	Studies employing dapagliflozin effectiveness in people with heart failure (HF) and related cardiovascular incidents.	Studies focusing on treatment, prognosis, or other aspects not related to dapagliflozin.
Outcome Measures	Studies providing specific outcomes measures (e.g., mortality rate, hospitalizations for heart failure, cardiovascular events, and changes in functional status and quality of life).	Studies lacking clear outcome measures.

Data Extraction

Each study was rigorously assessed during the data extraction process, particularly regarding participant characteristics, procedure complicity, and methodological uniqueness. To guarantee the presence of uniformity in data collection, a standard format was developed purposefully. The gathered information was then rewritten in an integrated manner to produce a succinct narration that outlined the major findings from different research projects. On top of that, a quantitative data analysis was also included to find out the efficiency of different management styles. This systematic way of understanding the data made it a lot easier to see what the similarities, differences, and patterns actually were. This was true even for all the research that was part of the study.

Quality Assessment

The Newcastle-Ottawa scale, which was employed for the overall scores, indicates that the reviewed literature has different levels of quality. Three main components that make up the evaluation are criteria of selection, compatibility, and outcome. The study yielded some interesting insights indicating that most of the articles transform from strong to average in these dimensions, but some of them have a combination of strengths and weaknesses. This provides a more holistic look into the whole of the data for this systematic review. Table 2 represents the quality assessment process.

**Table 2:** Representation of quality assessment process. Selection methods: Excellent: Demonstrates rigorous selection methods with low risk of bias; satisfactory: adequate selection methods with some risk of bias; inadequate: poor selection methods with a high risk of bias. Comparability: Excellent: High level of comparability between study groups; satisfactory: moderate comparability with some differences between study groups; inadequate: poor comparability between study groups. Outcomes: Excellent: Clear, relevant, and well-defined outcomes; satisfactory: acceptable outcomes but with some limitations in clarity or relevance; unsatisfactory: poorly defined or irrelevant outcomes.

Study ID	Selection methods	Comparability	Outcomes
Yeoh et al., 2020 [1]	Excellent	Excellent	Excellent
Vardeny et al., 2022 [2]	Excellent	Satisfactory	Unsatisfactory
McMurray et al., 2019 [4]	Satisfactory	Excellent	Excellent
McMurray et al., 2019 [6]	Excellent	Excellent	Satisfactory
Desai et al., 2022 [11]	Excellent	Satisfactory	Satisfactory
McMurray et al., 2019 [12]	Excellent	Satisfactory	Satisfactory
Kato et al., 2019 [13]	Excellent	Excellent	Satisfactory

Data Analysis and Synthesis

Using a narrative text approach, the authors analyzed and synthesized the data with an emphasis on dapagliflozin's safety and effectiveness in HF patients, with particular attention to its impact on mortality, CV events, HF hospitalization, and other relevant endpoints.

Results

Study Selection Process

**Figure 1:**
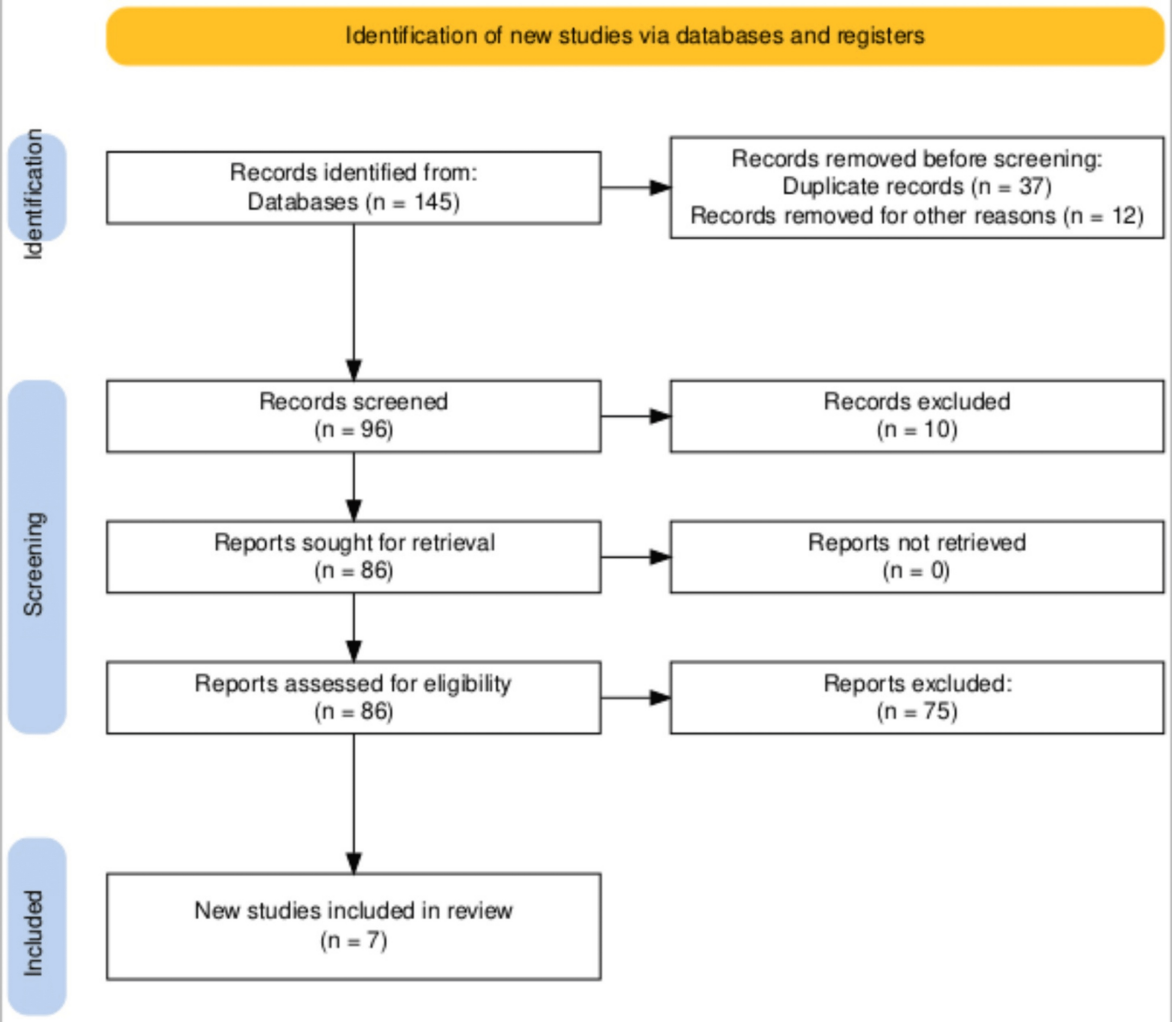
PRISMA flow diagram representing the study selection process.

After a thorough meticulous selection procedure, the electronic search generated 145 documents which were later narrowed down to 86 publications after removing articles that were duplicated or were not directly related to the research topic. The remaining 96 articles were reviewed for title/abstract, with 86 publications selected for full-text screening. Out of which, 79 reports were excluded as 28 reports were published before 2019, 16 were in other languages and 35 were not directly relevant. Figure 1 depicts the comprehensive screening procedure and the rationale behind exclusion using the PRISMA method.

Characteristics of Selected Studies

The characteristics of the seven studies that make up this review are displayed in Table 3. Considering the content's relevance to the study's goals, every article that was included in the analysis was of high quality. Each of them went into great detail to discuss the study's goal.

**Table 3:** Representation of the study selection process.

Study ID	Study design	Patient population	Follow-up duration	Intervention	Comparator	Sample size	Age (years)	Gender (%)
Vardeny et al., 2022 [1]	Randomized controlled trial (RCT)	Heart failure symptoms with a left ventricular EF greater than 40%	NA	10 mg dapagliflozin daily	Placebo	Total of 6,263 participants, 1,151 (18%) had HFimpEF	NA	NA
Yeoh et al., 2020 [2]	Randomized, double-blind, placebo-controlled, event-driven trial	Patients with heart failure (HF) and reduced ejection fraction (HFrEF) who are at least 18 years old, functional class II to IV in the New York Heart Association (NYHA) for at least two months, left ventricular ejection fraction (LVEF) ≤40%, elevated natriuretic peptide level, and receiving the best possible medication and device therapy for HFrEF	18.2 months	Dapagliflozin 10 mg once daily added to standard care	Placebo	4,744 patients	The mean age in the HF >5 years group was 68.1 years.	NA
McMurray et al., 2019 [4]	Phase 3, placebo-controlled trial	Individuals having an ejection fraction of 40% or less land heart failure classified as NYHA class II, III, or IV	Median of 18.2 months	Dapagliflozin (at a dose of 10 mg once daily) in addition to recommended therapy	Placebo in addition to recommended therapy	Dapagliflozin group (n=2,373), Placebo group (n=2,371)	Mean age of 66.2 years in the dapagliflozin group, 66.5 years in the placebo group	Approximately 24% female and 76% male in both groups
McMurray et al., 2019 [6]	Randomized, double-blind, controlled trial	Individuals suffering from reduced ejection fraction chronic heart failure (HFrEF)	360 days	Dapagliflozin 10 mg once daily in addition to standard care	Placebo in addition to standard care	4,744 patients	Mean age 66 years	23%women and 77% male participants
Desai et al., 2022 [11]	Randomized clinical trials	Individuals suffering from heart failure (HF) ranging in ejection fraction (EF)	NA	Dapagliflozin (10 mg, once daily)	Placebo	11,007 patients in the pooled analysis	Mean age provided 71.7 years	Males (70.0%) and females (30.0%)
McMurray et al., 2019 [12]	Randomized controlled trial (RCT)	Individuals suffering from reduced ejection fraction heart failure (HFrEF)	8 months	Dapagliflozin	Placebo	NA	NA	NA
Kato et al., 2019 [13]	Randomized, double-blind, multinational cardiovascular outcome trial	Individuals diagnosed with type 2 diabetes mellitus (T2DM) who have a creatinine clearance of at least 60 mL/min and either numerous risk factors or existing atherosclerotic cardiovascular disease (ASCVD)	Median of 4.2 years	10 mg dapagliflozin	Placebo	17,160 patients	NA	NA

Through the combined appraisal of the above-mentioned studies in Table 3, the complete yet complex influence of Dapagliflozin on HF patients is communicated in further depth. Interestingly, dapagliflozin, an SGLT2 inhibitor, has attracted attention recently and several RCTs have so far been conducted in distinguishing groups of patients and treatment options.

The main Phase 3 trial in HF patients' class II, III, and IV with HFrEF had placebo control, used to test patients' performances. These data have shown that only those patients who took the prescribed dosage of dapagliflozin along with the recommended treatment achieved better clinical improvement compared to placebo within a median follow-up of 18.2 months [4]. Yet, the qualities of the placebo and dapagliflozin groups in before-treatment characteristics were similar in mean age (around 66 years) and gender distribution (approximately 24% female and 76% male), this permitted us to maintain the balance of comparison.

Investigation into the cause-specific mortality across the range of EF leads to an even clearer Mansadlimens' influence in the bigger HF population. Bearing in mind the great number of those enrolled - 11,007 participants, among whom there were randomized clinical trials - the research showed that dapagliflozin may lead to a decrease in death rates in cases irrelevant to EF. A lower average age of participants was reported at 71.7 years in addition to a minor gender distribution with males (70.0%) accounting for a slightly higher proportion of the total while females (30.0%) were for a lesser proportion [11].

DAPA-HF, a trial, directly addressed patients with HF and a decreased EF in a double-blind placebo-controlled setting, calling for further validation of dapagliflozin’s efficacy. In the course of a 360-day timeframe, when dapagliflozin was administered as an additional drug to regular care at 10 mg per day, a significant decrease in negative outcomes was noted for the dapagliflozin group compared to placebo [6]. The age distribution of study subjects in this trial is quite similar to that of our previous study (around 66 years old), with a female-male ratio proportionate to it.

Similarly, together with the trials, the drug proved to have greater prospects in the management of HF during the different disease phases. The dapagliflozin trial performed in anemic HF and reduced left ventricular dysfunctions patients for at least two months throughout an 18-month follow-up regimen confirmed the significant benefits compared to placebo treatment [2]. The substudy of patients with HF duration over five years was sort of a kind of elucidating the multifaceted implications of dapagliflozin in this patient group with a mean age of 68.1 years.

Besides, if the results shown regarding the sample size and the patient characteristics do not show clarifying findings; the prespecified analysis of the DELIVER trial was properly designed to evaluate dapagliflozin only in patients with HF and an EF of more than 40% [1]. Early trials with dapagliflozin have allowed a new form of understanding as to the effectiveness of this drug therapy in a relatively uncharted subgroup of HF patients.

As can be deduced from these trials, dapagliflozin provides an exceptional showing of improvement, starting from the point of onset of clinical HF to the most advanced complications. Results from these trials essentially demonstrated the dapagliflozin efficacy to make it a standard in the HF treatment leading to reduced morbidity and mortality across the patient population. It is, nonetheless, vital for more studies involving long-term follow-ups to gain an in-depth understanding of how dapagliflozin works and what its maximum benefits can bring into the hospitalization of HF therapy. Table 4 shows primary and secondary outcomes along with HR (95% CI) and Interaction p-value.

**Table 4:** Primary and secondary outcomes along with HR (95% CI) and Interaction p-value.

Study ID	Primary outcome	Secondary outcome	HR (95% CI)	Interaction p-value
Vardeny et al., 2022 [1]	Increasing heart failure or cardiovascular death	Cardiovascular death, overall worsening heart failure events, and first worsening heart failure events	For HFimpEF patients, HR for primary endpoint was 0.74 (95% CI = 0.56–0.97)	Interaction p-value for treatment effect in HFimpEF subgroup was 0.43
Yeoh et al., 2020 [2]	Likelihood of cardiovascular death or increasing heart failure	A number of secondary endpoints, including as heart failure hospitalization or cardiovascular mortality, heart failure hospitalisations, alterations in the Kansas City Cardiomyopathy Questionnaire's total symptom score, and the frequency of declining renal function outcome and all-cause death.	NA	NA
McMurray et al., 2019 [4]	Combination of increasing heart failure and cardiovascular death	Composite of hospitalization for heart failure or cardiovascular death, total symptom score on the Kansas City Cardiomyopathy Questionnaire, composite of worsening renal function, death from any cause	NA	NA
McMurray et al., 2019 [6]	Reduction in incidence of worsening heart failure episode or cardiovascular death	Composite of heart failure hospitalization or cardiovascular death	NA	NA
Desai et al., 2022 [11]	Decrease in the composite rate of cardiovascular (CV) cause death or worsening HF events	NA	For CV death: HR = 0.86; 95% CI, 0.75-0.98 For sudden death: HR = 0.84; 95% CI, 0.70-1.01 For HF death: HR = 0.88; 95% CI, 0.70-1.11	NA
McMurray et al., 2019 [12]	Decrease in the frequency of heart failure or cardiovascular mortality events when dapagliflozin is used instead of a placebo.	Various secondary endpoints related to cardiovascular outcomes, heart failure events, renal function, patient-reported outcomes, etc.	NA	NA
Kato et al., 2019 [13]	Heart failure hospitalization or cardiovascular death	Cardiovascular death, all-cause mortality	NA	NA

Table 4 demonstrates primary and secondary outcomes, hazard ratios (HR) along CI with p-values from a variety of clinical trials that were performed to detect dapagliflozin effectiveness in people with HF and related CV incidents. These measures, which shed light on the valuable effects of dapagliflozin regardless of how the trials are being conducted and what the patient backgrounds are, clarify the medicinal benefits of this medication.

In the primary outcomes, several fight studies included composite endpoints which were the collective measures of worsening HF or CV event death. Dapagliflozin has shown a significant decrease in such composite outcomes, ranging from the placebo reference approximately 0.74 to 0.88 HR, which are supported by 95% confidence intervals as well particularly to estimate the treatment effect's precision [1,11]. Furthermore, interaction p-values were checked to investigate a possible differential treatment effect between groups that did respond differentially to treatment, even though not every trial reached statistical significance.

Secondary outcomes were contingent upon a varied CV event tackle, which included hospitalization due to HF, sudden cardiac death, and symptom scores for the patients. Short-term dapagliflozin treatment retained effects on reducing episodes of HF edema, CV death, and combined ex-FH endpoints. Of importance, such leading parameters are not only of value as part of the overall overview of dapagliflozin’s impact on CV disease management, but they can also be used to properly quantify this [2,11,13].

Thereby, the subgroup analyses also showed that dapagliflozin operated more efficiently in patients with HFpEF which is a group of HF patients. Specifically, within the subpopulations that were assigned to dapagliflozin, there was an improved treatment efficacy demonstrated through the reduction of HR for primary composite endpoints. Facilitated by interaction p-values, the assessment of whether subgroups have equal effects from the treatment or whether the difference is clinically significant is possible, which further assists in the labeling of effective treatment stratification strategies [4,6].

Contrary to this, the overall result will be enhancing the durability of the data obtained from the combined analysis of the primary and secondary outcomes and associated HR and interaction p-values. The abovementioned results expand the assets that are available in the literature of dapagliflozin being added to the treatment guideline in HF, which offers valuable outputs for the patients who are in the unfortunate situation of being impacted by it. Further research work is to be undertaken to establish the drug dapagliflozin mechanisms of action and to determine its optimal clinical utilization in HF patient diverse populations. Table 5 summarizes the study findings and adverse events.

**Table 5:** Summary of the study findings and adverse events.

Study ID	Findings	Adverse Event
Vardeny et al., 2022 [1]	Dapagliflozin reduced cardiovascular death or worsening heart failure in HFimpEF patients similarly to those with LVEF >40%. Subgroup analyses showed no significant difference in dapagliflozin's efficacy between HFimpEF and LVEF >40% groups. Patients with HFimpEF had a higher symptom burden initially but experienced improvements with dapagliflozin.	Diabetic ketoacidosis and serious adverse events related to volume depletion
Yeoh et al., 2020 [2]	Various findings from the study, including patient demographics, comorbidities, HF characteristics, background therapy, outcomes in relation to HF duration, and effects of dapagliflozin according to HF duration.	Volume depletion, renal events, major hypoglycemic events, bone fractures, diabetic ketoacidosis, amputation, and any diagnosis of Fournier gangrene.
McMurray et al., 2019 [4]	Diuretic and hemodynamic effects. Impact on myocardial metabolism, ion transporters, fibrosis, adipokines, vascular function.	Volume depletion and severe renal adverse events.
McMurray et al., 2019 [6]	Emphasizes the implementation of recommended heart failure therapies, particularly in patients with diabetes, and suggests incremental benefits of dapagliflozin beyond standard therapy for HFrEF patients.	NA
Desai et al., 2022 [11]	Reduction in CV death rates, particularly sudden death and demise from progressive HF, regardless of EF	NA
McMurray et al., 2019 [12]	Various potential mechanisms discussed, including diuretic-hemodynamic actions, impact on cardiac metabolism, adipokines, myocardial fibrosis, etc.	Volume depletion, renal dysfunction, major hypoglycemic episodes, fractures, diabetic ketoacidosis, amputations
Kato et al., 2019 [13]	Dapagliflozin reduces HHF in both HFrEF and non-HFrEF patients, but it significantly reduces cardiovascular death and all-cause mortality only in HFrEF patients. There are substantial absolute risk reductions in HFrEF patients treated with dapagliflozin. Dapagliflozin demonstrates a consistent safety profile across HF subgroups.	In patients with HF with reduced ejection fraction (HFrEF), there are no discernible increases in volume depletion events, acute renal failure events, diabetic ketoacidosis, or amputations linked to dapagliflozin therapy.

Table 5 illustrates the advantages and side effects of dapagliflozin drug in the failure of heart patients, which reveals how safe and effective the drug is, and what potent mechanisms it uses. They provide valuable information to help medical practitioners make informed decisions about HF treatment as well as the enhancement of care in HF patient management.

The results postulate that dapagliflozin has conspicuously positive effects on CV death rates, which is most obvious in the form of sudden death and mainly defined by HF development, regardless of the EF. The drug dapagliflozin exhibits extra HF benefits eclipsing the standard therapies setting aside the option of combining the drug with other HF medications, especially for patients with low EF.

So mechanistically like diuretics, hemodynamic actions, modulation of cardiac metabolism, ion transporters, fibrosis, adipokines, and vascular function, dapagliflozin’s effects cover numerous pathways. Besides, these complex machinations explain any positive outcomes in the trials [2,4,13].

When considering the adverse events reported, dapagliflozin displays a generally consistent safety profile across the HF subgroups with no cases of volume depletion events, acute renal failure, or diabetic ketoacidosis reported. There is also no indication of an increase in the rates of amputations in patients with HFrEF. Such being said, certain adverse events of importance, including the same realm of serious adverse events as volume depletion and renal events, will need to be closely observed.

On subgroup analyses, especially in patients with HF with improved EF (HFimpEF), it is apparent that dapagliflozin can show significant CV death/HF decompensation reduction with benefits swiped horizontally across patients with EF >40% [1]. The data in question indicate that the role of dapagliflozin in a range of HF types is an area for further investigation. These observations add more weight to dapagliflozin as a crucial therapeutic option.

To mention another safety issue, the standardized structure of adverse event reporting is restricted to the reporting of significant events, including fatal events leading to the suspension of treatment, volume reduction-related events, renal function complications, severe hypoglycemic episodes, fractures, diabetic ketoacidosis, and amputations. The set reporting approach not only helps in holistic assessment by conducting clinical trials but also lays the ground for preventive measures involving medication safety.

According to the comparison of advantages and disadvantages of dapagliflozin, it is seen that this is the finest drug of addition therapy for T2Dm, HF dyssynchrony, and RV remodeling. It is imperative that we strive to undertake more studies to discover their efficacy, safety, and acceptability as one of the diverse components of patient treatment. Last, but not least, it is the duty of the health services and pharmaceutical companies to shed light on the long-term effects of SGLT2 inhibitors and ensure the public health and patient outcomes of the apparatus of disease management.

Discussion

HF is a prevalent and severe condition, affecting over 64 million people worldwide, and contributing to significant morbidity and mortality rates across all regions [1]. Despite advancements in therapeutic interventions, there remains a gap in the development of treatment regimens that can comprehensively address the diverse needs of HF patients and reduce the global disease burden [6]. Recent clinical studies have demonstrated the effectiveness and safety of dapagliflozin, a SGLT-2 inhibitor, in improving outcomes in this population. This systematic review consolidates findings from these studies to evaluate dapagliflozin’s impact on HF management, highlighting its potential as a key therapeutic agent.

Social determinants of health, including socioeconomic status, education level, and access to healthcare, play a critical role in influencing HF outcomes [2]. These factors may act as confounders in evaluating the true effectiveness of interventions like dapagliflozin, as they could affect access to treatment, adherence, and long-term outcomes. Incorporating the influence of social factors into future studies will provide a more nuanced understanding of HF management and help tailor interventions to address health inequities.

The primary and secondary outcomes from various trials also indicate the effectiveness of dapagliflozin in reducing CV events in HF patients, as demonstrated across multiple trials. Notably, dapagliflozin exhibits a significant reduction in CV death rates, with the majority of deaths attributed to sudden cardiac death or progressive HF, irrespective of EF (EF) [4,6]. Subgroup analyses further highlight that dapagliflozin is effective not only in HFpEF but also in HF with improved EF (HFimpEF), suggesting its applicability across various HF phenotypes [1]. This study aligns with previously published reports, indicating that dapagliflozin can serve as an additional therapy in the management of HF, playing an important role in cardioprotective therapy.

Dapagliflozin has a broad range of beneficial actions, which are related to diuretic and hemodynamic mechanisms, and regulation of myocardial energy metabolism, ion transporters, fibrosis, adipokines, and vascular function. Such multidirectional processes create a basis for the improvement of cardiac function, prevention of HF exacerbation, and suppression of adverse CV events [11]. Discovering the mechanisms and working principles is therefore significant for the accomplishment of the drug's therapeutic effect and fine-tuning the HF treatment with dapagliflozin.

When it comes to safety, dapagliflozin has a generally positive profile. Among HF patients with reduced EF, there is no rise in the frequency of events such volume depletion, acute renal failure, diabetic ketoacidosis, etc. [1,2,11-13]. Adverse events reporting unified through the standardized system ensures thorough safety monitoring and influences the clinical practice such as using dapagliflozin in HF.

Clinically, the clinical data show that dapagliflozin should probably be included in the HF treatment strategies, especially in the patients with reduced LVEF. This highlights the necessity of not only following HF institutionally recommended therapy but also the introduction of dapagliflozin as an additional strategy in the quest for optimum outcomes. Moreover, subgroup analyses show that dapagliflozin is successful in a variety of patients, referring to its broad population usage for HF across the spectrum.

While the systematic review has encouraging results, the study incorporates several limitations at this point. A variety of trial designs, populations included, and outcome measures may lead to heterogeneity and thus to a limited power of comparison between different studies. We need long-term data about the direct application to clinical practice from dapagliflozin while supporting findings from clinical trials.

## Conclusions

Dapagliflozin has proven to be an excellent therapeutic option for HF patients, offering significant reductions in adverse CV events, including CV death, HF hospitalizations, and improvements in patient health across various HF phenotypes such as HFrEF, HFpEF, and HFimpEF. Its mechanisms of action, including diuretic and hemodynamic effects and its influence on myocardial metabolism, fibrosis, and vascular function, underscore its cardioprotective benefits. Moreover, dapagliflozin’s favorable safety profile, with low risks of adverse events like volume depletion or renal dysfunction, makes it a versatile option for a broad range of HF patients, including those with comorbid conditions like diabetes.

The incorporation of dapagliflozin into standard HF management protocols has the potential to improve patient outcomes and reduce the financial burden on healthcare systems by decreasing hospitalizations and long-term management costs. Although the evidence is compelling, further long-term studies are necessary to assess its impact across different HF subpopulations and its potential synergy with other therapies. As research progresses, dapagliflozin is likely to become a cornerstone of HF treatment, optimizing both clinical outcomes and healthcare efficiency.
